# A Microfluidic System for Real-Time Monitoring and In Situ Metabolite Detection of Plasma-Enhanced Wound Healing

**DOI:** 10.3390/biom15081077

**Published:** 2025-07-25

**Authors:** Zujie Gao, Jinlong Xu, Hengxin Zhao, Xiaobing Zheng, Zijian Lyu, Qiwei Liu, Hao Chen, Yu Zhang, He-Ping Li, Yongjian Li

**Affiliations:** 1 Department of Mechanical Engineering, Tsinghua University, Beijing 100084, China; gzj20@mails.tsinghua.edu.cn (Z.G.); xjl21@mails.tsinghua.edu.cn (J.X.);; 2 Department of Engineering Physics, Tsinghua University, Beijing 100084, China; 3 School of Clinical Medicine, Tsinghua University, Beijing 100084, China; 4 School of Basic Medical Sciences, Tsinghua University, Beijing 100084, China

**Keywords:** wound healing, cold atmospheric plasma, microfluidic chip, multiparametric sensing, metabolic dynamic monitoring

## Abstract

Although cold atmospheric plasma (CAP) has shown promise in facilitating wound repair due to its non-thermal and non-invasive properties, its dynamic effects on cellular response and metabolic regulation remain poorly characterized, and the mechanism is still unclear. In this study, we developed a microfluidic experimental system that integrates a CAP treatment module with multiparametric in situ sensing capabilities, along with precise environmental control of temperature, humidity, and CO_2_ concentration. A stratified microfluidic chip was engineered to co-culture HaCaT keratinocytes and HSF fibroblasts. CAP treatment was applied within this platform, and the dynamic processes of cell migration, proliferation, and multiple metabolic markers were simultaneously monitored. The experimental results show that the system can not only achieve real-time observation in the healing process under plasma intervention, but also find that the healing process is closely related to the concentration of NO_2_^−^. In addition, the study also found that keratin KRT14, which is thought to be closely related to wound healing, decreased significantly in the process of plasma-induced healing. The platform provides high-resolution experimental tools to elucidate the biological effects of CAP and has the potential for parameter optimization, material evaluation, and personalized therapeutic development to advance plasma research and clinical translational applications.

## 1. Introduction

Skin wound healing is a complex, multi-stage biological process involving inflammation, cell migration, re-epithelialization, and tissue remodeling, the efficiency of which directly affects regenerative outcomes and recovery time in patients [1]. To better understand the regulatory mechanisms underlying these processes, various experimental models have been developed, including two-dimensional (2D) cell cultures, animal models, and organoid systems [2,3]. While 2D monolayer cultures are widely used due to their simplicity and reproducibility, they fail to recapitulate the three-dimensional architecture and local microenvironment of real tissue. Animal models more closely mimic physiological conditions but are constrained by long experimental cycles, ethical considerations, and species-dependent variability, and offer limited control over microenvironmental and single-factor perturbations [4,5].

Conventional models often rely on endpoint sampling and offline analyses, lacking the capability for high-resolution, spatiotemporal monitoring of the wound healing process [6]. Studies have shown that key metabolic and signaling pathways involved in healing are highly time-sensitive; for example, inflammatory cytokine levels fluctuate dynamically, and reactive oxygen species (ROS) and glucose metabolism may exert opposing effects at different healing stages [7,8]. Real-time tracking of these molecular dynamics is thus critical for elucidating the mechanistic basis of wound repair, yet current platforms generally lack the capacity for in situ, multiparametric monitoring.

In response to these challenges, microfluidic technology has been increasingly applied to wound healing research in recent years. Its ability to construct three-dimensional microenvironments at the microscale, dynamically simulate cellular behaviors, and support high-throughput screening has positioned it as a promising experimental platform [2,9]. However, most existing microfluidic wound models still suffer from limited functional integration. On the one hand, wound generation methods often rely on mechanical or enzymatic approaches, which lack morphological precision and may disrupt the extracellular matrix, thereby compromising the accuracy of cell migration assessment [10]. On the other hand, many platforms focus primarily on cellular-level parameters such as migration rate and wound area, while failing to simultaneously monitor molecular-level signals such as metabolites and inflammatory factors [11].

Moreover, current systems typically operate using a single sensing modality—such as optical imaging or electrochemical detection—which constrains their ability to capture structural, functional, and metabolic information in parallel. This significantly limits their suitability for investigating complex exogenous stimuli such as CAP, which exerts multifaceted and time-sensitive biological effects [5]. There is thus an urgent need for an experimental system that integrates robust microenvironmental control with multimodal sensing capabilities, enabling real-time, multiparametric analysis of the full wound healing process and associated metabolic responses.

Cold atmospheric plasma has emerged as a promising modality in wound care due to its non-thermal nature, strong antimicrobial activity, and excellent biocompatibility [12]. Studies have shown that CAP generates reactive oxygen and nitrogen species (RONS) capable of modulating the local redox environment, stimulating cell migration and proliferation, and promoting angiogenesis and re-epithelialization [13,14]. However, these biological effects are highly time- and dose-dependent, influenced by multiple parameters such as treatment duration and discharge settings [15]. Prior to clinical translation, a mechanistic understanding of CAP’s action requires dynamic and quantitative experimental platforms [16]. Current research remains largely reliant on endpoint assays and static comparisons, lacking systems with the spatiotemporal resolution and multimodal detection capacity needed to capture dynamic cellular and metabolic responses to CAP treatment [17].

In this study, we developed a microfluidic experimental system that integrates CAP stimulation with in situ, multiparametric sensing, along with precise environmental control of temperature, humidity, and CO_2_ concentration. The platform incorporates optical, electrochemical, and fluorescence-based detection modules. Validation experiments demonstrated stable environmental conditions and continuous culture of skin cells with sustained viability above 90%. A microneedle-induced scratch model was established to simulate wound injury. Upon CAP treatment, the wound area contracted by approximately 28.6% within 12 h, with markedly enhanced cell migration and proliferation. This system offers a robust platform for real-time investigation of CAP-enhanced wound healing mechanisms and can be extended to high-throughput screening of treatment parameters, biomaterials, or combination therapies, thereby supporting data-driven development of personalized therapeutic strategies.

## 2. Materials and Methods

### 2.1. Development and Functional Testing of the Microfluidic Environmental Control System

The microfluidic platform used in this study integrates temperature, humidity, and carbon dioxide (CO_2_) regulation units to establish a stable and controllable cell culture environment. The main structure consists of a sealed incubation chamber with a transparent glass window on the top for real-time microscopic observation, and side ports for gas and electrical connections linked to an embedded control system and gas supply module. Temperature regulation is achieved by thin-film heating elements attached to the outer surface of the glass window, in combination with thermistor sensors that continuously monitor the internal temperature. The system is configured to operate within a range of 22.8–45.0 °C, with a PID algorithm enabling automatic heating. Temperature data are recorded at 1 min intervals to evaluate heating kinetics and thermal stability. Humidity is controlled by heating a water-saturated sponge to generate vapor, which is introduced into the chamber. A humidity sensor positioned centrally monitors the internal relative humidity, with a target setpoint of 97% RH. The regulation process is automatically logged by the system. CO_2_ concentration is controlled using a mass flow controller to regulate the input of premixed gas. The target concentration ranges from 1% to 19%, with real-time monitoring via an infrared CO_2_ sensor at a sampling interval of 5 s. All sensor outputs and control signals are integrated via an embedded data acquisition module and managed through a LabVIEW-based interface, which enables parameter adjustment, real-time visualization, and data recording.

### 2.2. Fabrication of the Microfluidic Skin-on-a-Chip Device

The skin-on-a-chip device was constructed from three layers of polydimethylsiloxane (PDMS), mimicking a bilayer epidermis–dermis architecture. It included upper and lower cell culture channels separated by a porous PDMS membrane. Each layer was fabricated using SU-8 photoresist molds. PDMS prepolymer (Sylgard 184, Dow Corning, Michigan, USA; 10:1 *w/w*) was degassed under vacuum, cast into the molds, and cured overnight at 65 °C. Following oxygen plasma treatment (120 s, 95% pure oxygen), the three layers were aligned and irreversibly bonded in a clean environment to ensure microchannel integrity and sealing. The upper PDMS layer (2 mm thick) contained a 10 mm central circular opening for gas exchange and manual access, along with 200 μm-wide parallel microchannels and two 1 mm ports for fluid circulation. The middle membrane (40 μm thick) contained uniform pores of approximately 5 μm diameter, allowing molecular exchange while preventing cell migration between layers. The lower PDMS layer (3 mm thick) mirrored the upper layer’s channel geometry. After assembly, the entire chip was sterilized by immersion in 75% ethanol followed by 2 h of UV exposure. To enhance cell adhesion, 50 μg/mL fibronectin (Procell, Wuhan, China) was injected into the microchannels and incubated at 37 °C for 1 h.

HaCaT keratinocytes and human skin fibroblasts (HSF, SV40-immortalized; Procell, Wuhan, China) were used to reconstruct the epidermal and dermal compartments, respectively. Prior to seeding, cells were cultured in T25 flasks using MEM or DMEM complete media (10% fetal bovine serum and 1% penicillin–streptomycin; Procell, Wuhan, China) until 70–90% confluence. Cells were harvested with 0.25% trypsin-EDTA at 37 °C for 5–15 min, followed by centrifugation at 900 rpm for 5–10 min. The resulting pellets were resuspended in prewarmed media for further use. HSF cells were seeded into the lower channel at a density of 0.5 × 10^6^ cells/mL (200 μL per well), followed by supplementation with HSF medium. The chip was incubated overnight at 37 °C in a humidified 5% CO_2_ incubator. The following day, the upper channel was rinsed three times with PBS to remove residual debris before seeding HaCaT cells at a density of 2 × 10^6^ cells/mL (200 μL per well). Equal volumes of HSF medium were used for both layers to maintain a consistent microenvironment. After an additional 24 h of incubation, both cell types exhibited attachment and spreading within their respective channels. To assess cell morphology and distribution, HaCaT and HSF cells were pre-labeled with CytoTrace™ CMTPX red fluorescent dye (AAT Bioquest, Pleasanton, CA, USA). Imaging was performed using an inverted fluorescence microscope. ImageJ software (version 1.51j8) was used to analyze fluorescence intensity, cell coverage, and spatial distribution, providing quantitative evaluation of cell viability, density, and uniformity.

To evaluate HaCaT cell attachment and proliferation within the chip, fluorescence images were acquired at 4 h, 12 h, 24 h, and 48 h post-seeding. Images were processed in ImageJ using threshold segmentation and binarization, and cell-covered area was calculated based on pixel count and converted to physical units using a calibrated scale (128 pixels = 100 µm).

The viability of cells after seeding into the chip was evaluated using the trypan blue exclusion assay. Cells were first enzymatically detached from the chip using 0.25% trypsin-EDTA. The resulting cell suspension was then mixed with 0.4% trypan blue solution at a 1:1 (*v*/*v*) ratio and incubated briefly for staining. The stained suspension was gently loaded into a hemocytometer chamber (0.1 mm^3^), and viable and non-viable cells were distinguished under a light microscope based on dye uptake. Cell viability was calculated as the percentage of unstained (live) cells relative to the total number of cells.

### 2.3. Construction of the On-Chip Wound Model

To establish a physical epidermal injury model in the microfluidic skin-on-a-chip, manual scratching was performed on the HaCaT cell layer using standard microneedles. Needles with diameters of 20 μm and 50 μm (Aladdin, Shanghai, China) were used to create scratch wounds of different widths. The procedure was conducted 24 h after seeding, once cells had adhered and spread uniformly. All operations were performed under sterile conditions. After exposing the upper channel, a sterilized needle was vertically applied to the cell layer, drawn along the main axis of the channel at a constant speed to form a linear scratch. After scratching, sterile phosphate-buffered saline (PBS; Procell, Wuhan, China) was gently added to the upper channel and flushed three times to remove detached cells and debris. Then, pre-warmed and pre-mixed complete media (1:1 *v/v*%, HaCaT complete medium and HSF complete medium) were added. Chips were returned to a humidified incubator at 37 °C with 5% CO_2_ for continued culture. The wound area was quantified by bright-field imaging using an inverted microscope. Images were imported into ImageJ software, and the scale was calibrated. The wound boundary was outlined manually or by threshold-based segmentation to calculate the two-dimensional projected area (A, μm^2^). For each image, 3–5 regions were randomly selected for measurement, and the average was calculated. All images were acquired using identical exposure and resolution settings to ensure consistency.

To visualize the wound morphology and cellular distribution, HaCaT cells were stained with CytoTrace™ CMTPX red fluorescent dye (AAT Bioquest, Pleasanton, CA, USA). Fluorescence microscopy was used to assess the extent of cell loss and spreading around the wound area. Additionally, the structural integrity of the porous PDMS membrane between channels was examined to ensure that the scratching procedure did not cause mechanical damage, preserving vertical diffusion and exchange functions essential for subsequent repair assays.

### 2.4. Plasma Treatment and Wound Healing Evaluation

After wound model construction for 24 h, CAP treatment was performed to evaluate its effect on cell migration and wound closure (Supplementary Material Figure S1). A floating-electrode dielectric-barrier-discharge (FE-DBD) plasma generator was used as described in Reference [18]. The system operated at a peak voltage of 5 kV and a frequency of 12 kHz. Water was used as the discharge medium, with the electrode inserted approximately 1 mm below the liquid surface. No external gas input was required. During treatment, the electrode was positioned above the wounded region in the upper channel of the chip, ensuring complete coverage of the wound area. Three treatment durations were applied: 0 min (control), 1 min, and 1.5 min. Immediately after discharge, fresh culture medium was replenished, and the chips were returned to a humidified incubator at 37 °C with 5% CO_2_.

Wound healing was monitored by fluorescence imaging at 0, 3, 6, 9, and 12 h post-treatment. Red fluorescent images of the wound area were acquired using an inverted fluorescence microscope. The wound closure was quantified by calculating the relative wound area percentage, denoted as φ, according to the following formula:

(1) $$\varphi =\frac{A_{h}}{A_{0}}\times 100\%,$$

where *A_h_* is the area of the wound at time *h*, and *A*_0_ is the area of the wound at the initial moment (*h* = 0). Each group included three replicates, and mean values were used for further statistical analysis. This quantitative index was used to assess the healing process and the effectiveness of CAP treatment.

### 2.5. Cell Proliferation and Tissue Repair Assessment

To evaluate the physiological changes in cells in the skin-on-a-chip after cold atmospheric plasma treatment, cell metabolic activity and the secretion level of repair-associated factors were analyzed. Cell viability was assessed using a colorimetric CCK-8 assay (Solarbio, Beijing, China). At 0, 6, 12, and 24 h after treatment, CCK-8 working solution was added to both upper and lower channels of the chip at 10% of the total medium volume. The chips were incubated at 37 °C for 2 h, then 100 μL of the reaction solution was collected and transferred into a 96-well plate. Absorbance at 450 nm was measured using a microplate reader. Each group included three replicate wells, and the measured absorbance values were used to evaluate cell metabolic activity. The secretion level of fibroblast growth factor 2 (FGF-2) was quantified using an enzyme-linked immunosorbent assay (ELISA). At 12 hours’ post-treatment, 100 μL of culture medium was collected from the upper channel of each chip and analyzed using a human FGF-2 ELISA kit (Solarbio, Beijing, China). Samples and standards (100 μL per well) were added to ELISA plates pre-coated with anti-FGF-2 antibodies, followed by incubation at 37 °C for 90 min. After discarding the liquid, wells were washed five times. Next, 100 μL of biotin-conjugated secondary antibody was added to each well and incubated at 37 °C for 1 h, followed by another wash step. Subsequently, 100 μL of horseradish peroxidase (HRP)-conjugated solution was added and incubated at room temperature for 30 min. After washing, 90 μL of TMB substrate solution was added and incubated in the dark for 15 min. Finally, 50 μL of stop solution was added to terminate the reaction. Absorbance at 450 nm was measured, and FGF-2 concentrations were calculated based on the standard curve.

### 2.6. Detection of Plasma-Generated Reactive Species

To evaluate the generation of reactive nitrogen species (RNS) following cold atmospheric plasma treatment, the concentration of nitrite (NO_2_^−^) in the culture medium was quantitatively measured using the Griess colorimetric assay. At 0, 3, 6, 9, and 12 h post-treatment, 100 μL of medium was collected from the upper channel of the chip. Each sample was mixed with an equal volume of Griess reagent (Solarbio, Beijing, China), incubated in the dark at room temperature in the dark for 10 min, and the absorbance was measured at 540 nm using a microplate reader. Nitrite concentration was calculated based on a standard curve, serving as an indicator of plasma-derived reactive species accumulation. Hydrogen peroxide (H_2_O_2_) generated during plasma treatment was also tested using Ampliflu Red (Aladdin, Beijing, China) combined with horseradish peroxidase (HRP; Aladdin, Shanghai, China). Ampliflu Red reacts with H_2_O_2_ under the catalysis of HRP to produce fluorescence, enabling the quantification of H_2_O_2_ distribution and concentration within a microfluidic skin wound model chip created by plasma. According to the manufacturer’s instructions, Ampliflu Red and HRP were diluted to appropriate working concentrations. Before plasma treatment, the solution containing Ampliflu Red and HRP was added to the on-chip wound model to ensure uniform coverage of the entire test area. After plasma treatment, fluorescence signal changes were observed immediately using a microscope. Ampliflu Red reacts with H_2_O_2_ to produce fluorescence under HRP catalysis, allowing the quantification of H_2_O_2_ generation and transport based on fluorescence intensity and distribution. WE obtained cell culture medium samples after plasma treatment, and detected the fluorescence intensity changes excited at 570 nm using an enzyme-linked immunosorbent assay (ELISA) reader to evaluate the generation of H_2_O_2_.

All measurements were performed in triplicate, and the results were used to assess plasma discharge intensity and the persistence of its biochemical effects.

### 2.7. Quantification of KRT14 Expression in HaCaT Cells by qPCR 

To investigate the potential molecular mechanisms by which cold atmospheric plasma promotes wound healing, the expression level of Keratin 14 (KRT14) in HaCaT cells was quantified using real-time quantitative PCR (qPCR). KRT-14 serves as a marker of basal keratinocytes, and its upregulation indicates epithelial activation and migratory responses during wound repair [19]. At 12 h and 24 h post-treatment, HaCaT cells were collected from the upper channel of the chip, rinsed with PBS, and lysed using Trizol reagent (TSINGKE, Beijing, China) for total RNA extraction. RNA purity was assessed by spectrophotometry, and samples with an A260/A280 ratio between 1.8 and 2.1 were selected for reverse transcription using a commercial cDNA synthesis kit (TSINGKE, Beijing, China). qPCR was performed using a SYBR Green PCR Master Mix (TSINGKE, Beijing, China) on an ABI 7500 real-time PCR system. Each 20 μL reaction contained specific primers for KRT14 (target gene) and GAPDH (reference gene). The thermal cycling conditions were as follows: 95 °C for 30 s (initial denaturation), followed by 40 cycles of 95 °C for 5 s and 60 °C for 30 s. Each sample was analyzed in triplicate. Relative gene expression was calculated using the 2^−ΔΔCt^ method to compare KRT14 levels at different time points and evaluate the molecular response to CAP treatment.

### 2.8. Statistics

All data are presented as means ± standard deviation (SD). Two-tailed unpaired Student’s *t*-test was used to assess the significance of differences. Statistical significance was defined as follows: * *p* < 0.05, ** *p* < 0.01, *** *p* < 0.001. The number of replicates performed for each experiment varied between three and eleven. The exact number is indicated in the corresponding figure legend.

## 3. Results

### 3.1. System Functional Testing

**Figure 1:**
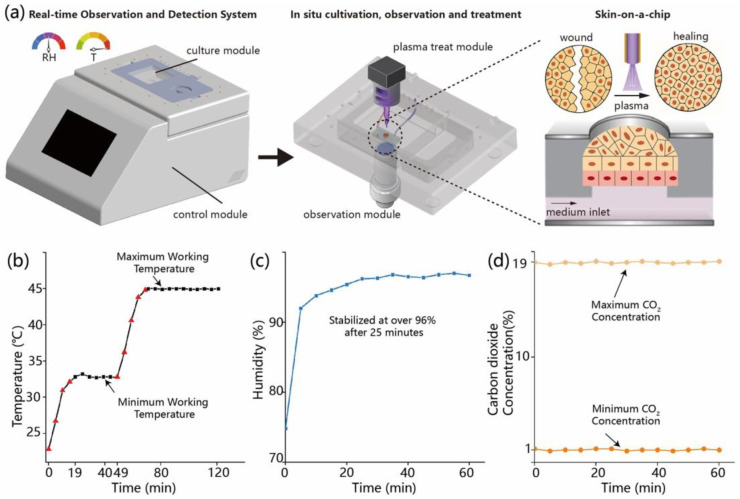
System testing. (**a**) Schematic diagram of the real-time observation and detection system, including the culture module, control module, plasma treatment module, and the skin-on-a-chip structure; (**b**) temperature control test results, showing temperature stabilization between the minimum and maximum working temperatures; (**c**) humidity control, demonstrating stabilization at over 96% after 25 min; (**d**) carbon dioxide concentration, illustrating the stable maximum and minimum CO_2_ levels during system operation.

We constructed a microfluidic system that enables continuous cell culture and real-time observation. Figure 1a shows the schematic design of the system and the chip structure with two types of cells. The operating temperature and temperature fluctuation test results are shown in Figure 1b. At a room temperature of 22.8 °C, the device reaches a minimum operating temperature of 32.8 °C. The system heats from room temperature to 32.8 °C within 19 min and subsequently maintains a stable temperature around 32.8 °C for 30 min, with fluctuations of less than 0.5 °C every 5 min. It then continues heating from 32.8 °C to the maximum operating temperature of 45 °C over 22 min and remains stable at approximately 45 °C for 120 min, again with temperature variations of less than 0.5 °C per 5 min interval. As shown in Figure 1b, red dots indicate the heating phase, while black dots represent the temperature stabilization phase.

The initial humidity in the incubator was 74.8%RH, as shown in Figure 1c. According to cell culture requirements, the humidity of the in vitro test system needs to be maintained above 90%RH for extended periods, so the system humidity was set to 97%RH (higher than 90%RH). The humidity reached 90% within 5 min and remained above this level for a prolonged duration, meeting the humidity requirements for continuous cell culture.

The initial CO_2_ concentration in the incubator was 1.3%, as shown in Figure 1d. The system CO_2_ concentration was set to the minimum value of 1%, and the CO_2_ concentration remained stable at 1% for 60 min (fluctuations were less than 0.2% every 10 min) to meet the minimum CO_2_ concentration requirements. The system was then adjusted to the maximum CO_2_ concentration of 19%, and it was found that the CO_2_ remained stable at 19% for 60 min (with fluctuations below 0.2% per 10 min interval), demonstrating good stability and compliance with the standard. The specific data are presented in Table 1.

### 3.2. Design, Construction, and Testing of Skin Chips

**Figure 2:**
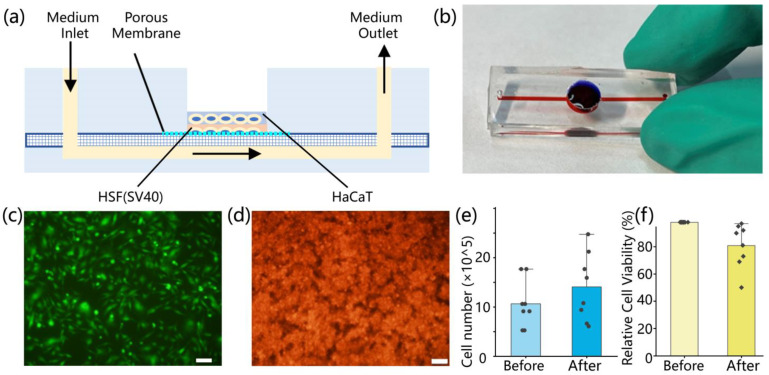
Skin-on-a-chip characterization. (**a**) Schematic diagram of microfluidic chip of bilayer cells; (**b**) the microfluidic chip with (**c**) fluorescence observation of HSF (SV40) cells (scale 100 μm); (**d**) fluorescence observation of HaCaT cells (scale 100 μm); (**e**) changes in cell number, *n* = 8; (**f**) changes in cell viability, *n* = 8.

In order to simulate the physiological characteristics of skin trauma, a skin tissue microfluidic chip was designed and fabricated, which featured a multi-layer biomimetic structure, including epidermis and dermis, to more closely mimic the structure of real skin tissue. A microfluidic network was incorporated within the chip, as shown in Figure 2a,b.

Through fluorescence observation, it was observed that the HSF stained green by CytoTrace™ CMTPX (AAT Bioquest, Pleasanton, CA, USA) exhibited a distinct fusiform or irregular triangle, which is typical of fibroblast morphology, and high cell density, as shown in Figure 2c. At the same time, HaCaT cells were stained red with CytoTrace™ CMTPX (Figure 2d), and results showed that HaCaT cells formed a confluent and compact cell layer within the chip, establishing a bilayer skin-on-a-chip. The attachment and proliferation process of HaCaT cells is further detailed in Supplementary Figure S2a, which presents fluorescence images taken at 4 h, 12 h, 24 h, and 48 h post-seeding. The images demonstrate that the cells gradually spread and fused, indicating continuous proliferation within the chip. The total cell number increased from the initial 1.0 × 10^6^ to 1.405 × 10^6^ (Figure 2e), and the cell-covered area stabilized after 24 h (Supplementary Figure S2b). The cell viability of HaCaT cells showed a slight decrease before and after seeding, but remained at a relatively high level (Figure 2f). Some relatively low viability values may have resulted from incomplete enzymatic digestion of highly confluent cells before seeding, which led to the misjudgment of viable cells as dead by trypan blue staining. To ensure experimental consistency, only chip batches with viability above 80% at 4 h post-seeding were included in subsequent CAP treatment experiments. Additionally, Supplementary Figure S2c provides viability measurements of well-seeded skin-on-a-chip constructs at 12 h, 24 h, and 48 h, all above 85%, indicating a healthy cellular state and suitability for wound construction and CAP treatment studies.

### 3.3. Wound Construction and Evaluation

**Figure 3:**
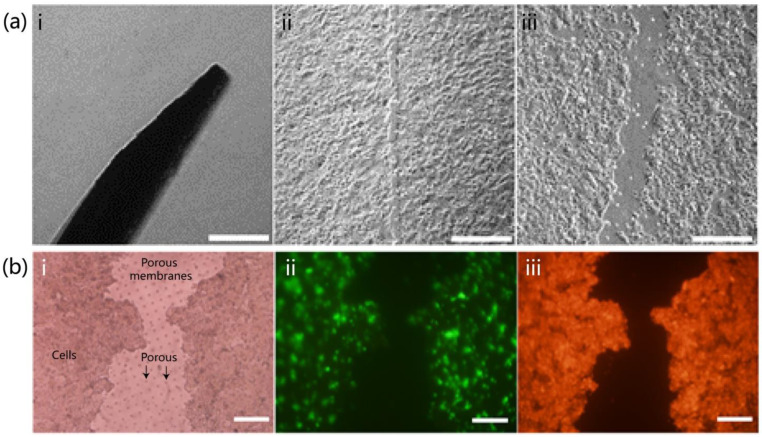
Trauma construction and evaluation. (**a**) Trauma was constructed using microneedles, with images showing (**i**) the microneedles used for trauma creation, (**ii**) minimal trauma, and (**iii**) maximal trauma (scale 100 μm). (**b**) Evaluation of post-traumatic cells: (**i**) brightfield image showing the membrane indicated by arrows as intact; (**ii**) fluorescence image of HSF (SV40) cells stained in green; (**iii**) fluorescence image of HaCaT cells stained in red (scale 100 μm).

Scratch wounds of varying widths were created using standard microneedles of different sizes. For example, a 20 μm microneedle produced a representative scratch, as shown in Figure 3a(i). This method yielded scratches with minimum and maximum widths of 18.9 ± 2.0 μm (Figure 3a(ii)) and 53.5 ± 8.4 μm (Figure 3a(iii)), respectively. Microscopic observation confirmed that the porous membrane in the middle layer of the chip remained intact after scratch formation (Figure 3b(i)), providing a substrate for subsequent cell proliferation. Fluorescence imaging showed HSF cells in green (Figure 3b(ii)) and HaCaT cells in red (Figure 3b(iii)), with a high degree of overlap between the two layers, indicating that the chip maintained its double-layer cellular structure after the scratch was introduced. Plasma treatment is applied to the surface after the wound is constructed, as described in Supplementary Material Figure S3.

### 3.4. Healing Effect and Mechanism of Plasma on Wounds

**Figure 4:**
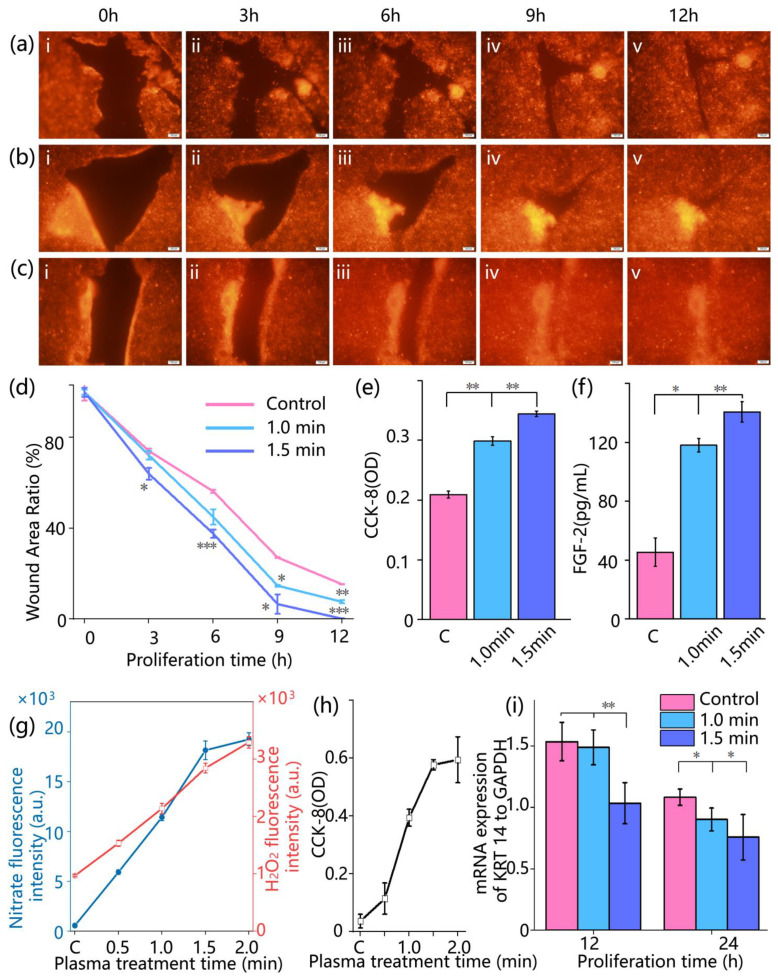
Fluorescence observation of wound healing process and study of the mechanisms of wound healing. (**a**) Control group, no CAP treatment, (**i**) 0 h; (**ii**) 3 h; (**iii**) 6 h; (**iv**) 9 h; (**v**) 12 h; (**b**) Plasma treatment for 1 min, (**i**) 0 h; (**ii**) 3 h; (**iii**) 6 h; (**iv**) 9 h; (**v**) 12 h; (**c**) plasma treatment for 1.5 min, (**i**) 0 h; (**ii**) 3 h; (**iii**) 6 h; (**iv**) 9 h; (**v**) 12 h. In (**a**–**c**), red represents HaCaT cells (**d**) change in wound area ratio over time, *n* = 3, * *p* < 0.1, ** *p* < 0.05, *** *p* < 0.01; (**e**) CCK-8 absorbance change, *n* = 3, ** *p* < 0.05; (**f**) FGF-2 absorbance change, *n* = 3, * *p* < 0.1, ** *p* < 0.05; (**g**) nitrate concentration and H_2_O_2_ concentration with plasma treatment time, *n* = 3; (**h**) the cell proliferation curves with plasma treatment time, *n* = 3; (**i**) KRT14 mRNA expression at 12 h and 24 h *n* = 3, * *p* < 0.1, ** *p* < 0.05.

As shown in Figure 4a, the wound recovers itself without any treatment. With increasing proliferation time, the wound area gradually decreased. However, the natural repair capacity remained limited. After 12 h, a clear unhealed wound was still visible (Figure 4a). Following 1 min of plasma treatment, the wound healing process was significantly accelerated. By 12 h, only a small unhealed area remained in the 1 min treatment group (Figure 4b). In the group treated with plasma for 1.5 min, wound closure was even more rapid. By 9 h, the wound had nearly healed, and by 12 h, no visible wound was detected under microscopic observation (Figure 4c).

To quantitatively assess the wound healing process and eliminate the influence of initial wound size, the wound area ratio φ was calculated. In the control group, φ_control_ (12 h) was 15.29% ± 0.058% (Figure 4d, red line). For the 1 min plasma treatment group, φ_1min_ (9 h) was 14.65% ± 0.420%, comparable to φ_control_ (12 h), and further decreased to φ_1min_ (12 h) = 7.54% ± 0.700%. In the 1.5 min treatment group, φ_1.5min_ (9 h) was 6.54% ± 4.281%, and φ_1.5min_ (12 h) reached 0.69% ± 0.101%, indicating nearly complete wound closure.

Furthermore, cell proliferation was evaluated using the CCK-8 assay, as shown in Figure 4e. At 12 h, the OD value in the control group was 0.21 ± 0.006. In comparison, the OD value increased to 0.30 ± 0.007 in the 1 min plasma treatment group (*p* = 0.00529) and to 0.34 ± 0.005 in the 1.5 min group (*p* = 0.0015), indicating a significant enhancement in cell proliferation following plasma treatment.

There was a significant increase in FGF-2 secretion following plasma treatment, as shown in Figure 4f. In the control group, the FGF-2 concentration in the culture medium was 45.33 ± 9.693 pg/mL. This value rose to 118.08 ± 4.544 pg/mL in the 1 min treatment group (*p* = 0.0107) and further to 140.68 ± 6.956 pg/mL in the 1.5 min group (*p* = 0.0077), indicating a dose-dependent enhancement of FGF-2 expression.

Moreover, to further explore the mechanism behind the observed enhancement in cell proliferation, we analyzed the production of reactive species. Plasma discharges generate typical reactive products mainly categorized into reactive nitrogen species (RNS) and reactive oxygen species (ROS). Among them, reactive nitrogen species dissolve in the aqueous phase to form stable nitrite ions, while hydrogen peroxide in reactive oxygen species is considered a key functional component. Therefore, these two representative products were selected to assess the concentration of reactive species in plasma.

As shown by the blue and red curves in Figure 4g, the nitrite and H_2_O_2_ concentrations gradually increased with longer plasma exposure, although the growth rate began to plateau after 1.5 min. Correspondingly, cell proliferation, indicated by the black curve, also increased with treatment time, as shown in Figure 4h. However, in the 2.0 min group, the CCK-8 absorbance was 0.349 ± 0.0198, nearly identical to that of the 1.5 min group (0.344 ± 0.00453), suggesting that prolonged exposure may lead to increased cytotoxicity, offsetting the proliferative effects.

Finally, the molecular-level effects of CAP treatment on wound healing were assessed by examining KRT14 mRNA expression, a marker of epithelial cell status. As shown in Figure 4i, at 12 h, the expression ratio of KRT14 to GAPDH was 1.23 ± 0.125 in the control group, 1.19 ± 0.114 in the 1 min treatment group—comparable to the control—and significantly lower in the 1.5 min group at 0.83 ± 0.134. By 24 h, KRT14 expression levels had declined across all groups, with values of 0.87 ± 0.054 (control), 0.72 ± 0.074 (1 min), and 0.61 ± 0.148 (1.5 min). These results suggest that KRT14 expression is regulated in a time- and dose-dependent manner during CAP-induced wound healing, with downregulation observed under more intensive treatment, potentially reflecting shifts in cellular differentiation or repair status. Wound recovery at 12 h and 24 h under microscope after 1 min, 1.5 min, and no treatment are shown in Supplementary Material Figure S4.

## 4. Discussion

This study presents an innovative microfluidics-based platform that integrates in situ cell culture, observation, and treatment, and demonstrates its application in investigating the biological effects of CAP on wound healing. By incorporating precise environmental control and continuous culture modules, this platform maintains stable conditions of temperature, humidity, and CO_2_, enabling long-term cell cultivation. Notably, the system supports in situ CAP treatment of skin-on-a-chip while preserving a sterile and stable environment. In addition, it facilitates continuous, real-time tracking and precise quantification of essential biological processes, including cell migration, proliferation, and metabolite production, via optical and colorimetric assays. This addresses a major limitation of conventional experimental approaches, where frequent environmental changes often distort tissue repair processes and obscure rapid biological responses. Consequently, this platform provides a powerful and high-resolution in vitro tool for in-depth, systematic investigation of CAP–biological system interactions, particularly in the context of wound healing, bridging a critical technological gap in the field.

One of the key advantages of this study lies in the successful construction and application of a biomimetic, double-layered skin-on-a-chip that replicates the structural and functional characteristics of native human skin with high fidelity. By co-culturing HaCaT keratinocytes and HSF fibroblasts on a porous membrane, the model reconstructs a skin architecture comprising epidermal and dermal equivalents, while simulating blood perfusion through microchannels. Compared with existing skin models [20], the advantage of this system is that it can observe the wound healing status in real time, which means that during the culture process, the cell chip does not need to be displaced at all. Meanwhile, cell proliferation, budding, and other phenomena can be directly observed, allowing continuous and undisturbed tracking of key biological processes. Meanwhile, we systematically investigated the biological effects of CAP on wound healing, which accelerated wound closure by promoting both cell migration and proliferation. Time-lapse imaging showed enhanced collective migration and more rapid wound coverage in the CAP-treated group compared to controls. This observation was further supported by CCK-8 assays (Figure 4e), which revealed increased cellular metabolic activity, and by the upregulation of FGF-2 secretion (Figure 4f), indicating a CAP-induced boost in proliferative signaling.

Moreover, this biological effect is driven by the synergistic actions of multiple ROS and RNS generated by CAP. Our study demonstrates that the concentrations of nitrite and hydrogen peroxide both increase within the cellular microenvironment, representing a stable byproduct of RNS and a key molecule of ROS, respectively [21]. Notably, these concentration levels exhibit a strong positive correlation with the rate of wound closure. We have demonstrated the critical role of RNS in tissue repair, with nitric oxide (NO), a major RNS component, effectively modulating inflammatory responses during wound healing and significantly promoting tissue regeneration [21]. In the present study, the enhanced healing effects observed following CAP treatment, along with elevated nitrite levels in the cellular microenvironment, provide further validation of these earlier findings.

In addition, this study examined the expression dynamics of KRT14, a key marker protein of keratinocytes [22]. Following CAP treatment, KRT14 mRNA levels exhibited a progressively accelerated downregulation within 24 h, which correlated with the enhanced wound closure rate. Importantly, this downregulation was not attributable to cytotoxic effects. On the contrary, CCK-8 assay results (Figure 4e) indicated significantly higher metabolic activity in the CAP-treated cells compared to the control group throughout the observation period. In the later phases of wound re-epithelialization, as wound closure progresses, basal keratinocytes begin to differentiate and migrate into the suprabasal layers. This transition is accompanied by a physiological downregulation of KRT14 expression, reflecting the onset of epidermal maturation and stratification [23,24]. Accordingly, the more pronounced and earlier reduction in KRT14 expression observed in the CAP-treated group, particularly in the 1.5 min treatment group, suggests that CAP may accelerate the transition of keratinocytes from the proliferative and migratory phase to the differentiation and maturation phase. This provides molecular-level evidence supporting the role of CAP in promoting faster wound healing.

Despite the progress made, this study has certain limitations that warrant further investigation. While the current study offers valuable insights, several limitations remain to be addressed. First, on the biological level, although the in vitro skin model developed here successfully mimics the epidermal–dermal architecture, it lacks key components such as immune cells and a fully representative extracellular matrix. As a result, it does not fully capture the physiological complexity of the in vivo wound microenvironment. [25]. Although HaCaT is a commonly used keratinocyte line [26,27], primary normal human keratinocytes might better reflect the human wound healing process [28], and can be considered for future research. Meanwhile, the number of replicates in some omics-related experiments was limited in this study, and future work should include larger sample sizes to improve statistical reliability [29]. In addition, biomarker analysis in this study employed methods appropriate to the characteristics of each target. For example, ELISA was used to quantify the secreted protein FGF-2, while qPCR was used to assess the gene expression of the intracellular structural protein KRT14. However, cross-validation of key biomarkers at both the protein and transcript levels would provide a more rigorous basis for mechanistic interpretation [30]. Moreover, this study focused primarily on the short-term biological effects of CAP treatment, whereas its impact on longer-term processes such as tissue remodeling remains to be elucidated [31].

The integrated microfluidic platform developed in this study offers substantial potential beyond its immediate application in wound healing research. The core design principles and methodologies are broadly applicable to other emerging biomedical areas involving cold atmospheric plasma, such as cancer therapy and antimicrobial treatment. Owing to its modular architecture, the platform enables high-throughput screening, which is essential for the systematic optimization of treatment parameters, the evaluation of novel biomaterials, and the identification of synergistic drug combinations. This versatility not only enhances the efficiency of fundamental research in plasma medicine but also provides a robust framework for the development of data-driven and personalized therapeutic strategies.

## 5. Conclusions

In conclusion, this study advances our understanding of CAP’s mechanisms in wound healing while showcasing the transformative potential of microfluidic technology in experimental biomedicine. By providing a high-resolution, dynamic view of cellular and molecular responses, the platform addresses longstanding challenges in plasma research and offers a robust tool for future investigations. While limitations remain, the integration of microenvironmental control, biomimetic design, and multiparametric sensing represents a significant step toward standardized, clinically relevant plasma therapies. Future work should focus on expanding the platform’s biological complexity and exploring its applications in other areas of plasma medicine to fully realize its potential.

## Figures and Tables

**Table 1:** System parameter test results.

Basic Parameters	Environmental Parameters	Test Results	
		Minimum	Maximum
Temperature (°C) ^1^	22.8	32.8	45
Temperature Fluctuations (ΔT)	/	±0.25	±0.05
Heating time (min) ^1^	/	41	
CO_2_ concentration	1.3%	1%	19%
Humidity	74.8%	>90.0%RH	

^1^ Heat from room temperature to the highest temperature.

## Data Availability

All data supporting the results reported in this study are available within the main text and Supplementary Materials of this article.

## Supplementary Materials

The following supporting information can be downloaded at: https://www.mdpi.com/article/10.3390/biom15081077/s1, Figure S1: Plasma treatment process; Figure S2: Attachment and viability of HaCaT cells in the skin-on-a-chip device; Figure S3: Time-flow diagram of cell seeding, culture, scratch construction, plasma treatment, healing, and detection; Figure S4: Wound recovery at 12 h and 24 h under microscope, after 1 min, 1.5 min and no treatment (scale 100 μm).

## Abbreviations

The following abbreviations are used in this manuscript:

ROS	Reactive oxygen species
CAP	Cold atmospheric plasma
RONS	Reactive oxygen and nitrogen species
PDMS	Polydimethylsiloxane
DBD	Dielectric barrier discharge
HRP	Horseradish peroxidase
RNS	Reactive nitrogen species
qPCR	Quantitative PCR
SD	Standard deviation
