# Supplementary material for: A Microfluidic System for Real-Time Monitoring and In Situ Metabolite Detection of Plasma-Enhanced Wound Healing
Source: Biomolecules. 2025 Jul 25;15(8):1077. doi: 10.3390/biom15081077 (PMC12383944; doi:10.3390/biom15081077)
Supplement: Supplementary file 1 [file biomolecules-15-01077-s001.zip › biomolecules-3704773-supplementary.pdf]

Supporting Information

A Microfluidic System for Real-Time Monitoring and In Situ Metabolite Detection of Plasma-Enhanced Wound Healing

Zujie Gao †1, Jinlong Xu †1, Hengxin Zhao [2], Xiaobing Zheng [1], Zijian Lyu [2], Qiwei Liu[1], Hao Chen [3], Yu Zhang [4], He-Ping Li [2,*] and Yongjian Li [1,*]

1 Department of Mechanical Engineering, Tsinghua University, Beijing 100084, China; gaozu-jiebuaa@163.com (Z.G.); xjl21@mails.tsinghua.edu.cn (J.X.)
2 Department of Engineering Physics, Tsinghua University, Beijing 100084, China
3 School of Clinical Medicine, Tsinghua University (Beijing Tsinghua Changgung Hospital), Beijing 100084, China
4 School of Basic Medical Sciences, Tsinghua University, Beijing 100084, China
* Correspondence: liheping@tsinghua.edu.cn (H.-P.L.); liyongjian@tsinghua.edu.cn (Y.L.);
Tel.: +86-13901018491 (H.-P.L.); +86-010-62772801 (Y.L.)
† Z.G. and J.X. contributed equally to this paper.

Contents **Page No.**

Figure S1. Plasma treatment process 2

Figure S2. Fluorescence microscopy image before HaCaT proliferation 2

Figure S3. Time-flow diagram of cell seeding, culture, scratch construction, plasma treatment, healing, and detection. 2

Figure S4. Wound recovery at 12h and 24h under microscope, after 1 min, 1.5 min and no treatment. 3

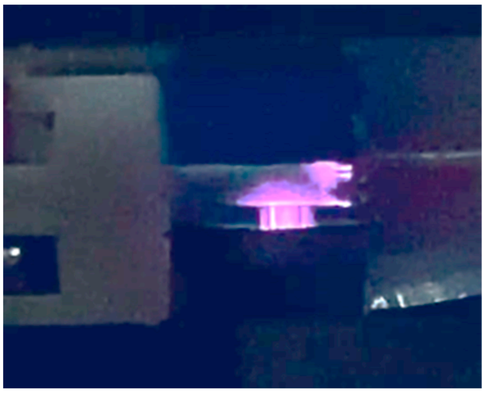

Figure S1. Plasma treatment process. The process of plasma treatment generates purple light by utilizing atmospheric medium for stable discharge, which results in the formation of purple sparks on the surface of the culture medium pool.

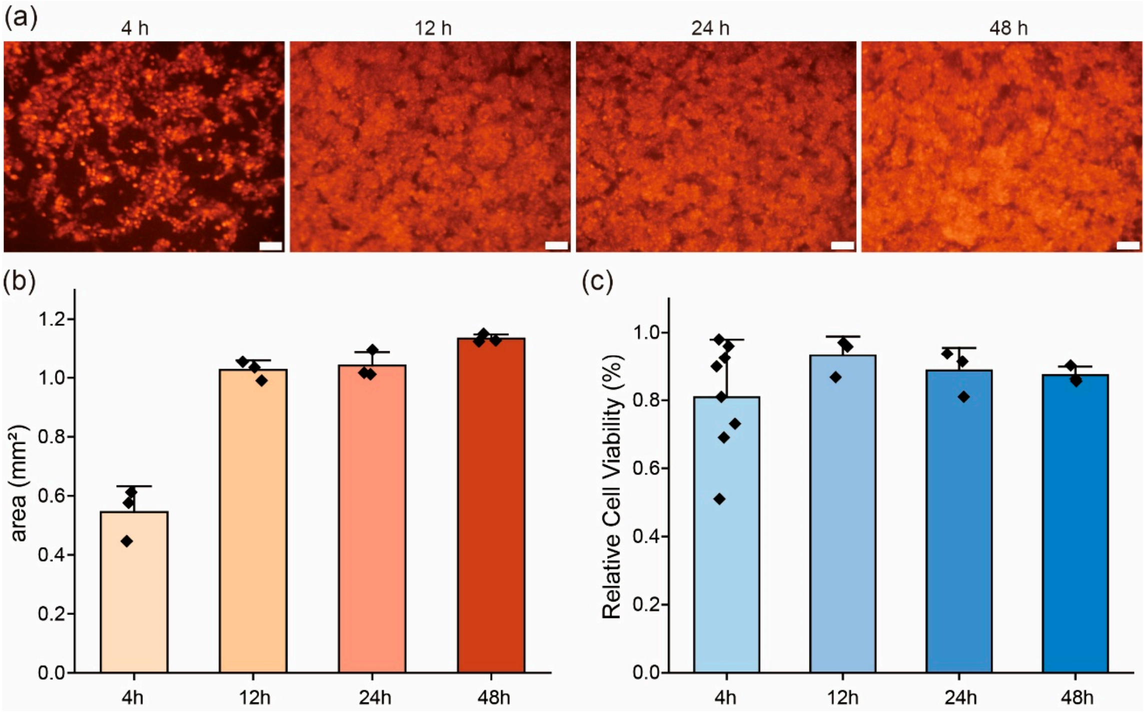

Figure S2. Attachment and viability of HaCaT cells in the skin-on-a-chip device. (a) Fluorescence images of HaCaT cells at 4 h, 12 h, 24 h, and 48 h after seeding, showing their spreading morphology and confluence. Cells quickly adhered and expanded over time. Scale bar: 100 µm. (b) Quantitative analysis of cell-covered area. Fluorescent regions were binarized and measured using ImageJ, and the pixel area was converted into actual physical area (mm^2) based on the scale (128 pixels = 100 µm). Results showed a rapid increase within the first 12 h, followed by plateauing, indicating good proliferation. (c) Relative cell viability at each time point (n = 8 for 4 h group, and n = 3 for other groups). Cells were detached 4 h after seeding and stained with trypan blue for manual counting. A slight decline in the 48 h group may result from stronger adhesion and reduced enzymatic dissociation. Overall, cell viability remained above 85%, demonstrating a healthy status suitable for subsequent experiments.

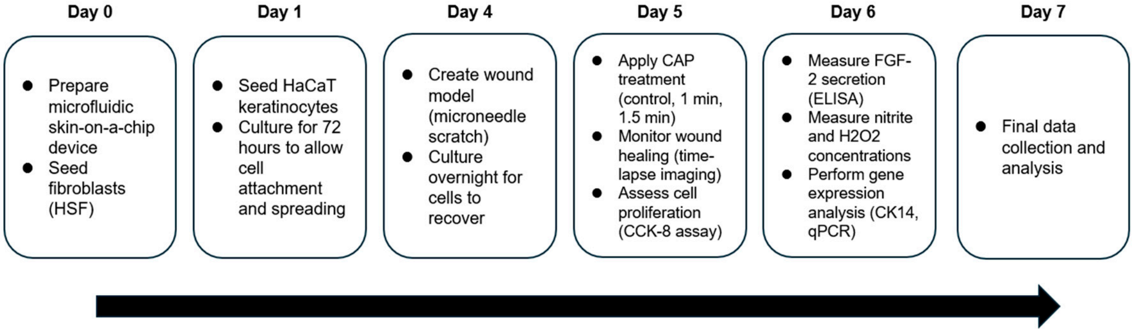

Figure S3. Time-flow diagram of cell seeding, culture, scratch construction, plasma treatment, healing, and detection. This diagram illustrates the sequential steps involved in the experimental process. Initially, HaCaT cells are seeded onto the chip, followed by a period of cell culture, allowing them to proliferate and settle. After sufficient growth, a scratch is introduced to simulate a wound on the cell monolayer. The plasma treatment is then applied for specific time intervals (1 min or 1.5 min), during which stable discharge produces purple sparks on the surface of the culture medium. Subsequently, healing progresses as the cells migrate to close the wound gap. Finally, detection methods are employed to monitor wound recovery at designated time points (12 h and 24 h) under the microscope, allowing for assessment of cell proliferation and wound healing dynamics.

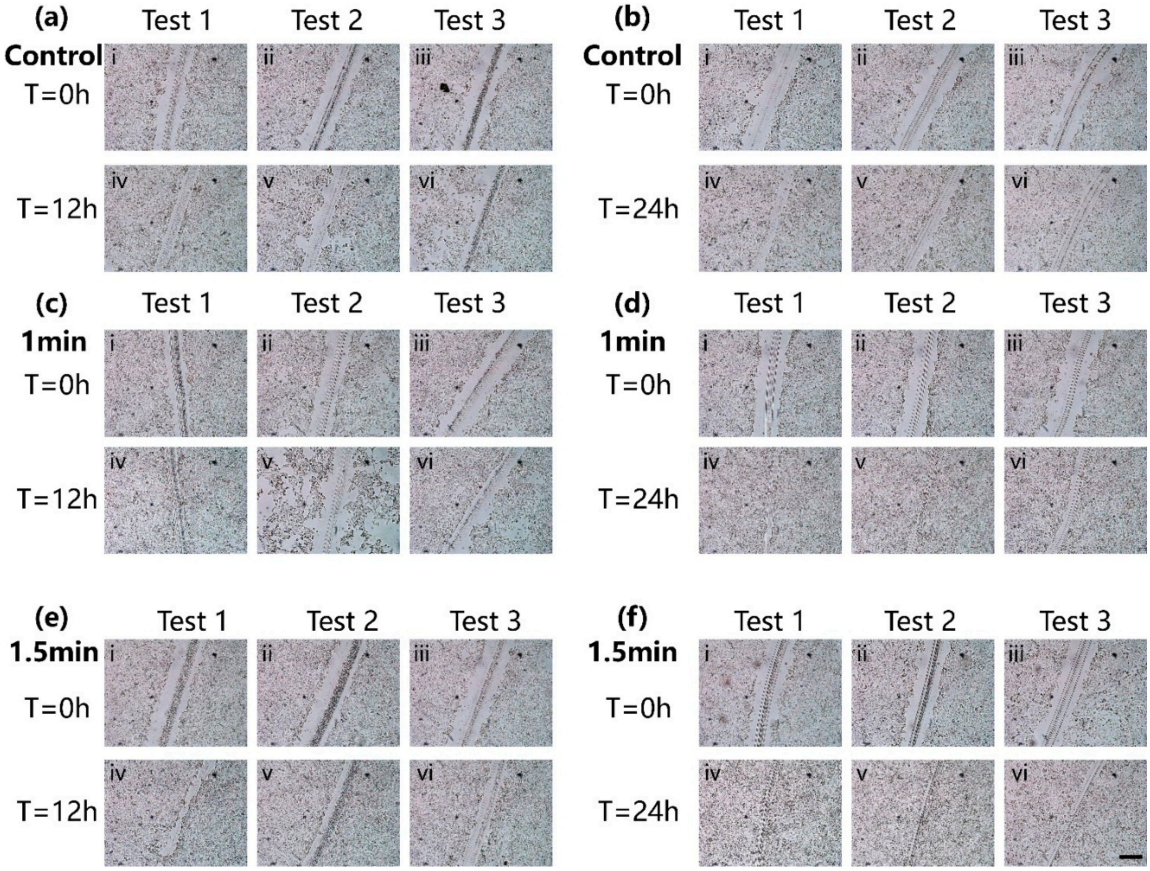

Figure S4. Wound recovery at 12h and 24h under microscope, after 1 min, 1.5 min and no treatment (scale 100μm). (a) the control group (no treatment) at 0 and 12 hours; (b) Wound recovery in the control group (no treatment) at 0 and 24 hours; (c) Wound recovery after 1 minute plasma treatment at 0 and 12 hours; (d) Wound recovery after 1 minute plasma treatment at 0 and 24 hours; (e) Wound recovery after 1.5 minute plasma treatment at 0 and 12 hours; (f) Wound recovery after 1.5 minute plasma treatment at 0 and 24 hours.
